# Combining e-nose and e-tongue for improved recognition of instant starch noodles seasonings

**DOI:** 10.3389/fnut.2022.1074958

**Published:** 2023-01-09

**Authors:** Rong Ma, Huishan Shen, Hao Cheng, Guoquan Zhang, Jianmei Zheng

**Affiliations:** Engineering Research Center of Grain and Oil Functionalized Processing in Universities of Shaanxi, College of Food Science and Engineering, Northwest A&F University, Xianyang, Shaanxi, China

**Keywords:** instant starch noodles seasonings, e-nose, e-tongue, combined data, multilayer perceptron neural networks analysis

## Abstract

Seasonings play a key role in determining sensory attributes of instant starch noodles. Controlling and improving the quality of seasoning is becoming important. In this study, five different brands along with fifteen instant starch noodles seasonings (seasoning powder, seasoning mixture sauce and the mixture of powder and sauce) were characterized by electronic nose (e-nose) and electronic tongue (e-tongue). Feature-level fusion for the integration of the signals was introduced to integrate the e-nose and e-tongue signals, aiming at improving the performances of identification and prediction models. Principal component analysis (PCA) explained over 85.00% of the total variance in e-nose data and e-tongue data, discriminated all samples. Multilayer perceptron neural networks analysis (MLPN) modeling demonstrated that the identification rate of the combined data was basically 100%. PCA, cluster analysis (CA), and MLPN proved that the classification results acquired from the combined e-nose and e-tongue data were better than individual e-nose and e-tongue result. This work demonstrated that in combination e-nose and e-tongue provided more comprehensive information about the seasonings compared to each individual e-nose and e-tongue. E-nose and e-tongue technologies hold great potential in the production, quality control, and flavor detection of instant starch noodles seasonings.

## 1. Introduction

Instant starch noodles are made from various starch (potato, bean, or cereal starch), water and other ingredients, and processed into dry, no-cooking, convenience food (1). It usually with the seasonings sprinkled over the starch noodles and are ready to eat after soaked in boiling water for 3–4 min. Along with the national health consciousness enhancement, fortification has become an accepted practice in order to improve the nutritional properties of instant starch noodles. Instant starch noodles can be fortified with seasonings or additives to improve their nutritional properties (2). For example, many additives such as chitosan and polysaccharide gum are used to improve the properties of starch noodles (3). Secondly, changing the processing method can improve its taste and quality characteristics. Compared with the traditional hot air drying and open sun drying, the heat-moisture treatment improved the hardness, springiness, and chewiness textural properties of starch noodles (4). Greenhouse drying improved the quality and sustainability of starch noodles (5). Research is still underway to produce instant starch noodles that are more nutritious and meet taste requirements.

Seasonings play a key role in determining sensory attributes of instant starch noodles. Seasonings are mainly used for improving aroma and taste. Aroma is an important sensory attribute of flavor in food and volatile compounds plays an important role in it (6). Nowadays, only few studies have examined the volatile compounds in instant noodle seasonings (7). Therefore, further studies on aroma of instant noodle seasoning are required to elucidate the characteristic volatile compounds. Instant starch noodles seasonings usually come as either seasoning powder or seasoning mixture sauce. The seasoning powder contains various spices, such as salt, yeast extract, sodium glutamate, disodium 5′-ribonucleotide, paprika powder, and pepper. The seasoning mixture sauce is made with refined vegetable oil, various spices, yeast extract, soybean paste, and bone broth. Instant starch noodles seasonings contain various amino acids, nucleotides, vitamins, and minerals.

Consumers are mainly concerned about the flavor of instant starch noodles and the major difference of flavors is the variety of instant starch noodles seasonings. Different brands of instant starch noodles seasonings with the same taste vary greatly in flavor. To a certain extent, brand is an indicator of the product’s quality and flavor. Different brands of instant starch noodles seasonings possessed variant chemical ingredients, aroma, taste, and price (8). Basically, the flavor of seasoning is evaluated by the experts through senses, however, the experts are expensive, time-consuming, and susceptible to the external environment and the interior factors of man himself, which leads to biased conclusions (9). Therefore, it is essential to establish a rapid and accurate technology to perceive the quality differences among different brands of instant starch noodles seasonings, which could also be used to detect the phenomenon of selling disqualified products at best quality prices.

Electronic nose (e-nose) and electronic tongue (e-tongue) are sensor-based apparatus which imitate the olfactory and gustatory perception of human. They provide fast detection and comprehensive information about the sample, and they can monitor and differentiate samples that are otherwise difficult for human sensory panels to discriminate (10). The e-nose and e-tongue are used to characterize small differences in taste and odor of food. They are powerful tools for distinguishing flavor characteristics and are widely used in food quality evaluation (11). Xu et al. (10) applied e-nose, e-tongue, and e-eye combined with three chemometrics methods to identify and predict the tea quality, results indicated that fusion signals outperformed the independent signals, could perfectly predict the contents of the main chemical components. Qiu et al. (12) employed e-nose and e-tongue to discriminate two types of Satsuma mandarins from different developmental stages and trace the change in internal quality. Yu et al. (13) and Yin et al. (14) used HS-SPME-GC/MS combined with e-nose and e-tongue to analyze the volatile profiles and taste properties of soybean paste and Harbin red sausages. However, to the best of our knowledge, there are few studies on the combination of e-nose and e-tongue to detect and analyze the comprehensive flavor profile of instant starch noodles seasonings.

The main objectives of this study was to establish an easy, rapid and objective identification method for instant starch noodles seasonings based on different flavor profiles. Therefore, we used e-nose and e-tongue independently and in combination to discriminate different brands of instant starch noodles seasonings. Then, recognition of the seasonings was performed by principal components analysis (PCA), cluster analysis (CA), and multilayer perceptron neural networks analysis (MLPN), with the intention of testing whether e-nose and e-tongue can provide a rapid and accurate approach for flavor recognition and quality assurance of instant starch noodles seasonings.

## 2. Materials and methods

### 2.1. Sample selection and preparation

Five different brands of instant starch noodles with braised beef were obtained at the commercial market in China. The five brands were marked as C, D, G, L, and Z, respectively. Each brand contained three categories: seasoning powder (P), seasoning mixture sauce (S), and the mixture of powder and sauce (M). Table 1 summarizes detailed information about the tested seasoning samples.

**TABLE 1:** Five brands of instant starch noodles seasonings samples.

	Seasoning powder (P)	Seasoning mixture sauce (S)	The mixture of powder and sauce (M)	Producing area
Samples	CP	CS	CM	Guangdong
	DP	DS	DM	Zhejiang
	GP	GS	GM	Sichuan
	LP	LS	LM	Jiangsu
	ZP	ZS	ZM	Anhui

### 2.2. E-nose analysis

The PEN3 portable e-nose (Airsense Analytics GmbH., Schwerin, Germany) was applied to classify and characterize the instant starch noodles seasonings. The apparatus is composed of a sampling unit and a gas detection system consisting of 10 metal oxide semiconductor (MOS) sensors with varying sensitivity to each volatile compound (15). Prior to analysis, the e-nose was preheated and calibrated with pure air.

Based on the preliminary experimental results, 7 g of seasoning powder, 4 g of seasoning mixture sauce, and 11 g of the mixture of powder and sauce were put into three 150 mL beakers, respectively. The seasoning solution was prepared after pouring 100 mL boiling water into the beaker and stirring for 2 min. A volume of 10 mL sample solution was put into a 30 mL sealed glass vial and incubated for 30 min at 40°C. The chamber and injection flow rate was 400 mL/min. The measurement time, sample interval time, flush time, and automatic zero adjustment time were 60, 1, 300, and 5 s. Each sample was measured 10 times.

### 2.3. E-tongue analysis

Instant starch noodles seasonings were analyzed using ASTREE e-tongue (Alpha M. O. S. Co., Toulouse, France). The instrument consists of an automatic sampler, chemical sensor array, and chemometric software package (16). The sensor array includes seven different chemical sensors and a reference electrode (Ag/AgCl) (17).

According to preliminary experiments, 6 g of seasoning powder, 8 g of seasoning mixture sauce, and 14 g of the mixture of powder and sauce were put into three 500 mL beakers, respectively. The seasoning solution was prepared after pouring 400 mL boiling water into the beaker and stirring for 2 min. The seasoning solution was first filtered using a piece of gauze and the filtrate was then filtered through a 0.45 μm nylon syringe filters. Then, 80 mL of filtrate of each sample was analyzed using the e-tongue. Before the test begins, 0.01 mol/L HCl, NaCl, and sodium glutamate were applied to activate, calibrate, and diagnose the chemical sensors (18). The e-tongue parameters were as follows: acquisition time of 120 s, clean time of 10 s, acquisition period of 1 s, stirring rate of 3 r/s. Each sample was measured in three times.

### 2.4. Combining the e-nose and e-tongue datasets

Data fusion involves different levels of data abstraction (19), with low-level fusion being the most common method and giving good results. In low-level fusion, the e-nose and e-tongue signals were simply concatenated before model construction. In the resulting data matrix, the number of rows is equivalent to the number of samples, and the number of columns is equivalent to the number of signals obtained from e-nose and e-tongue (20). We used this low-level fusion to combine e-nose and e-tongue data for the combined analyses discussed below.

### 2.5. Data analysis

The individual e-nose and e-tongue data and their fusion were applied to PCA, CA, and MLPN. PCA is the most popular feature extraction method in pattern recognition. It is used to differentiate samples and extract the most important information from data. PCA reduces the dimensionality of datasets while retaining most of the information of the original data (21). PCA interprets the data in two or three dimensions by the first two or three PCs, which explain the largest amount of variance in the data (22).

Cluster analysis is an unsupervised classification technology which can cluster samples according to their similarity in multidimensional space (23). It identifies degrees of homogeneity of samples that are similar, while samples placed in different clusters reflect dissimilarity (24). A hierarchical CA method was applied to classify 60 samples based on the response signals of 17 sensors (7 e-tongue sensors and 10 e-nose sensors), with the squared Euclidean distance taken as a measure of similarity and the Ward’s method taken as the amalgamation rule.

Multilayer perceptron neural networks analysis is a feed-forward supervised classification method consisting of an input layer, several hidden layers, and an output layer (22). It can be described as a non-linear projection vector from input to output. The sensors and different brands of instant starch noodles seasonings were used as the input layer and output layer, respectively. Training dataset used 10 sensors as inputs, eight neurons in the first hidden layer and six neurons in the second hidden layer. The four groups of different drop heights were used as the outputs, so there were four neurons in the output layer. The activation function of the hidden layer is a hyperbolic tangent function, and the activation function of the output layer is a Softmax function (25). Besides, to achieve a good generalization capability, the dataset used for adjusting the neural networks was again divided into a training set and a testing set. Again the 60 samples (12 samples for each group) were divided into two groups: 9 samples (75 percent of samples) for the training, and 3 samples (25 percent of samples) for the testing.

Principal component analysis and MLPN were performed using Statistical Product and Service Solutions (SPSS) 20.0. CA was conducted using Minitab 16.2.

## 3. Results and discussion

### 3.1. Response signals of e-nose

**FIGURE 1:**
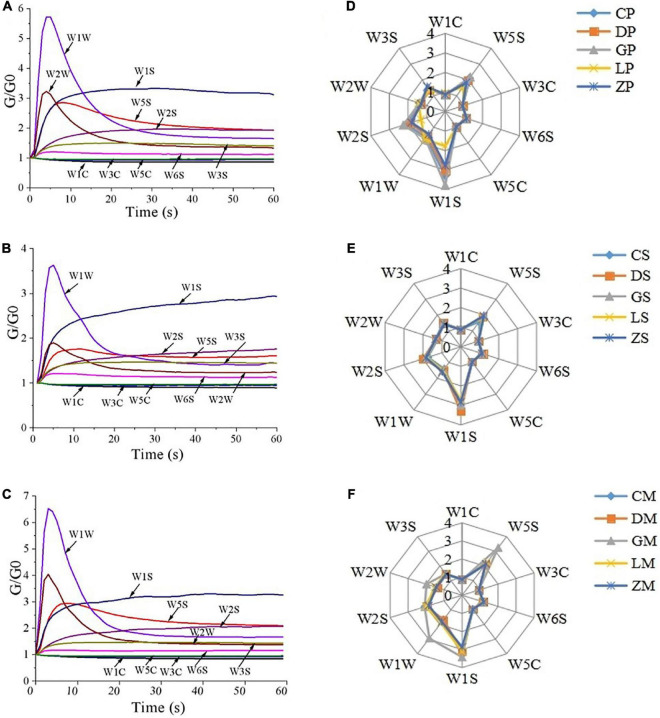
E-nose responses to instant starch noodles seasonings of CP **(A)**, CS **(B)** and CM **(C)**, and radar plots of 10 e-nose sensor responses for seasoning powder **(D)**, seasoning mixture sauce **(E)** and the mixture of powder and sauce **(F)**.

The typical e-nose response curves of CP, CS, and CM are shown in Figures 1A–C. The response signal was denoted by relative resistivity (G/G0), where G and G0 are the conductivities of the sensors when exposed to sample gas and pure air, respectively (26, 27). The e-nose response curves in the three figures exhibited similar temporal changes. The response values of sensors W1W, W2W, and W5S reached peak value at the 5th second and reduced quickly anaphase, finally, it reached a stable value. The response values of W1S rose rapidly from 0 to 10 s, then increased more slowly slightly, and finally remained high for the rest of the measurement. The response values of W3S, W2S, and W6S was appearing to increase slightly. In contrast, W1C, W3C, and W5C reduced slightly, where G/G0 values were generally less than one since they are negative metal-oxide sensors (28). Overall, the response signals of W1S, W2S, W5S, and W1W remained at a high level (Figures 1A–C). The results suggested that aromatic compounds, nitrogen oxides, terpenes, and sulfur-containing compounds were the main constituents in the aroma of instant starch noodles seasonings (29).

The radar plots of e-nose sensor responses are presented in Figures 1D–F. There were no significant differences among the 10 sensors in seasoning powder, seasoning mixture sauce, and the mixture of powder and sauce (except GM), indicating that all seasonings had similar aromas. The W1S, W2S, W5S, and W1W sensors had stronger responses than other sensors. These results were in accordance with the typical e-nose response curves (Figures 1A–C). The GP sample had higher values of W1S, W2S, and W5S than other samples, while LP had lower values of W1S, W2S, and W5S than other samples (Figure 1D). The DS sample had higher values of W1S and W2S than other samples (Figure 1E). Similarly, GM exhibited the highest response values in the sensors of W1S, W2S, W5S, W1W, and W2W (Figure 1F). The results suggested that different brands of instant starch noodles seasonings had significant difference in aromas.

### 3.2. Response signals of e-tongue

**FIGURE 2:**
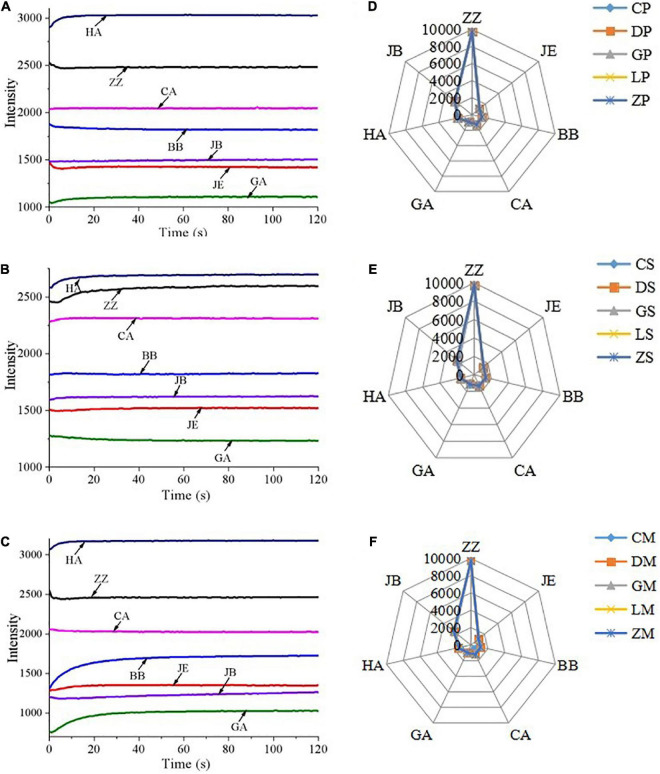
E-tongue responses to instant starch noodles seasonings of CP **(A)**, CS **(B)** and CM **(C)**, and radar plots of 10 e-tongue sensor responses for seasoning powder **(D)**, seasoning mixture sauce **(E)** and the mixture of powder and sauce **(F)**. ZZ, JE, BB, CA, GA, HA, and JB are seven different liquid cross-selection sensors for the electronic tongue.

The e-tongue sensor responses of CP, CS, and CM are shown in Figures 2A–C. For the seasoning powder, the signals of HA and GA increased from 0 to 10 s, while the signals of ZZ, BB, and JE decreased during this time period. These signals started to stabilize after approximately 30 s, and fully stabilized at 120 s. For the seasoning mixture sauce, the signals of HA, ZZ, and CA increased from 0 to 30 s, and other response sensors had no significant changes. These results suggested that HA, ZZ, BB, and GA are the main contributors to taste profiles of instant starch noodles seasonings.

The radar plots of e-tongue sensor responses are presented in Figures 2D–F, which show similar overall trends. Instant starch noodles seasonings of different brands cannot be distinguished by simply observing the response curves and radar charts of the samples. It is necessary to use multivariate statistical analysis to process the e-tongue data. Therefore, we used the stable signals at 120 s for PCA analysis.

### 3.3. Analysis of e-nose data

**FIGURE 3:**
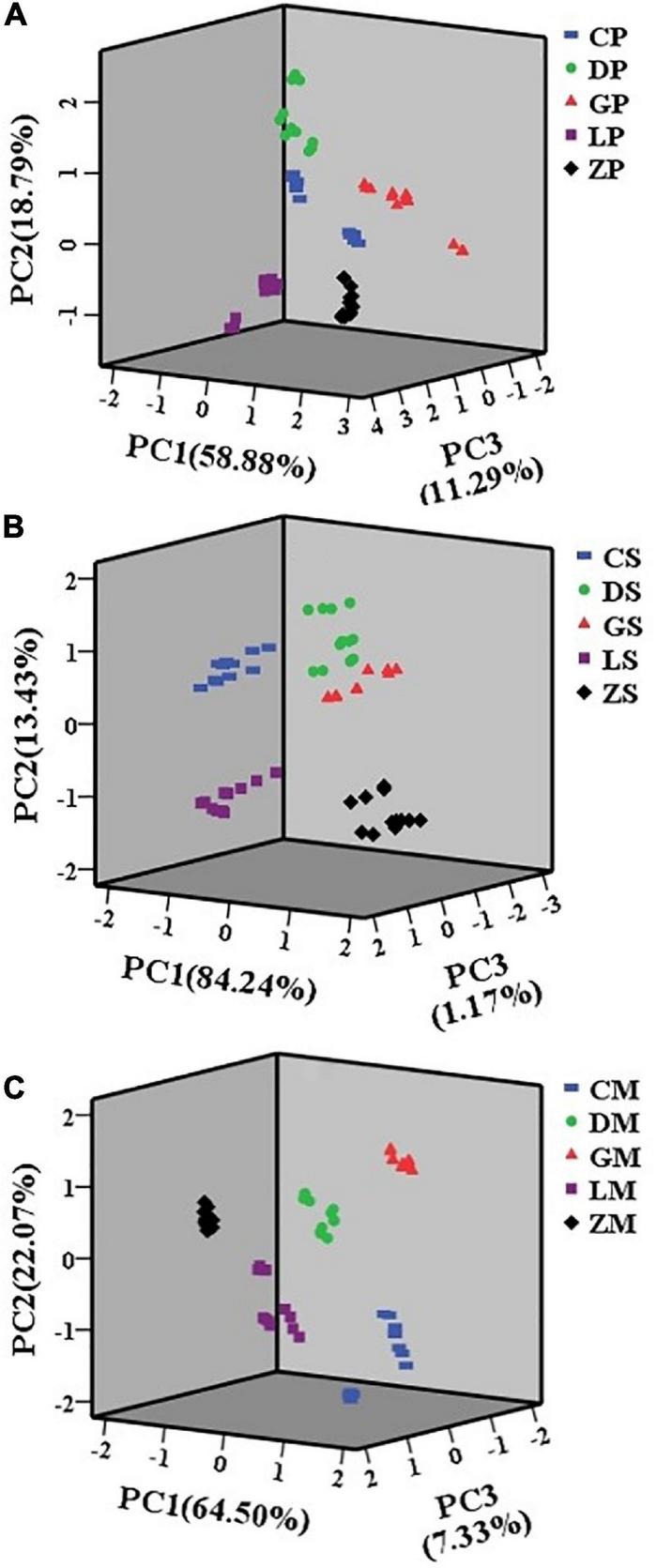
Principal component analysis (PCA) plots of seasoning powder **(A)**, seasoning mixture sauce **(B)**, and the mixture of powder and sauce **(C)** using e-nose.

The e-nose PCA plots are presented in Figure 3. The seasoning powder samples of the same brand were located together. PC1, PC2, and PC3 explained a combined 88.96% of the total variation. For the seasoning mixture sauce, the first three PCs explained 98.84% of the total variance. PC1 explained 84.24% of the total variance, and PC2 explained 13.43% of the total variance, PC3 explained 1.17% of the total variance. For the mixture of powder and sauce, PC1 accounted for 64.50% of the total variance. PC2 accounted for 22.07% of the total variance and PC3 accounted for 7.33% of the total variance. In total, the first three PCs explained over 85.00% of the total variance in e-nose data.

In multidimensional space, the samples were clustered into five non-overlapping groups. Samples with more similar flavor would appear closer to each other. The CP and DP samples were located very close to each other, suggesting that CP and DP had similar aromas. The characteristic aroma attributes of CS, DS, and GS were also similar. The results showed that principal components analysis of e-nose data can be used to classify different brands of instant starch noodles seasonings.

### 3.4. Analysis of e-tongue data

**FIGURE 4:**
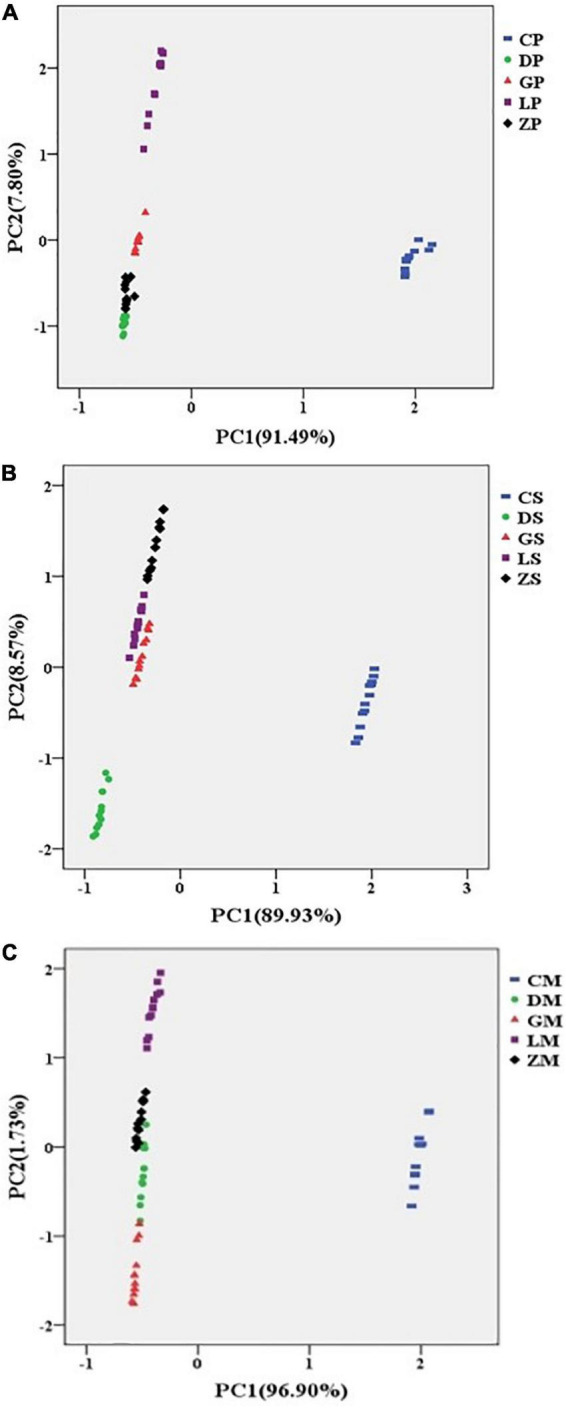
Principal component analysis (PCA) plots of seasoning powder **(A)**, seasoning mixture sauce **(B)**, and the mixture of powder and sauce **(C)** using e-tongue.

The e-tongue PCA plots are presented in Figure 4. For seasoning powder, 91.49% of the total variance was explained by PC1 and 7.80% of the total variance was explained by PC2. Similarly, for seasoning mixture sauce, the first two PCs explained 98.50% of the total variance among samples. For the mixture of powder and sauce, the first two PCs explained 98.63% of the total variation. Thus, for all three types of samples, PC1 and PC2 explained almost all the total variance.

As shown in Figures 4A–C, the CP, CS, and CM samples were distributed mainly on the positive side of PC1, while the rest of samples were mainly located in the negative side for PC1. The results indicated that the taste profiles of CP, CS, and CM were strikingly different from other samples. The possible reasons were that different manufacturers used different materials and production processes, which may cause the chemical ingredients and tastes of instant starch noodles seasonings to differ among brands. In general, the PCA results suggested that e-tongue can properly characterize different brands of instant starch noodles seasonings (30).

### 3.5. Analysis of combined e-nose and e-tongue datasets

**FIGURE 5:**
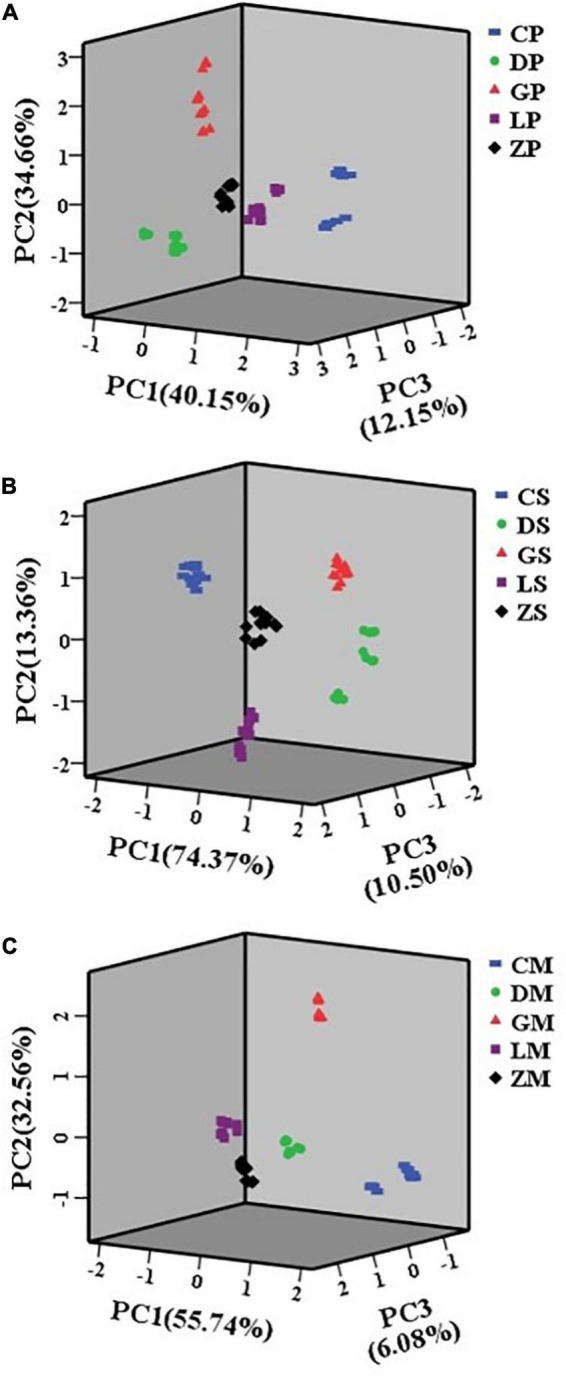
Principal component analysis (PCA) plots of seasoning powder **(A)**, seasoning mixture sauce **(B)**, and the mixture of powder and sauce **(C)** using combined data.

**FIGURE 6:**
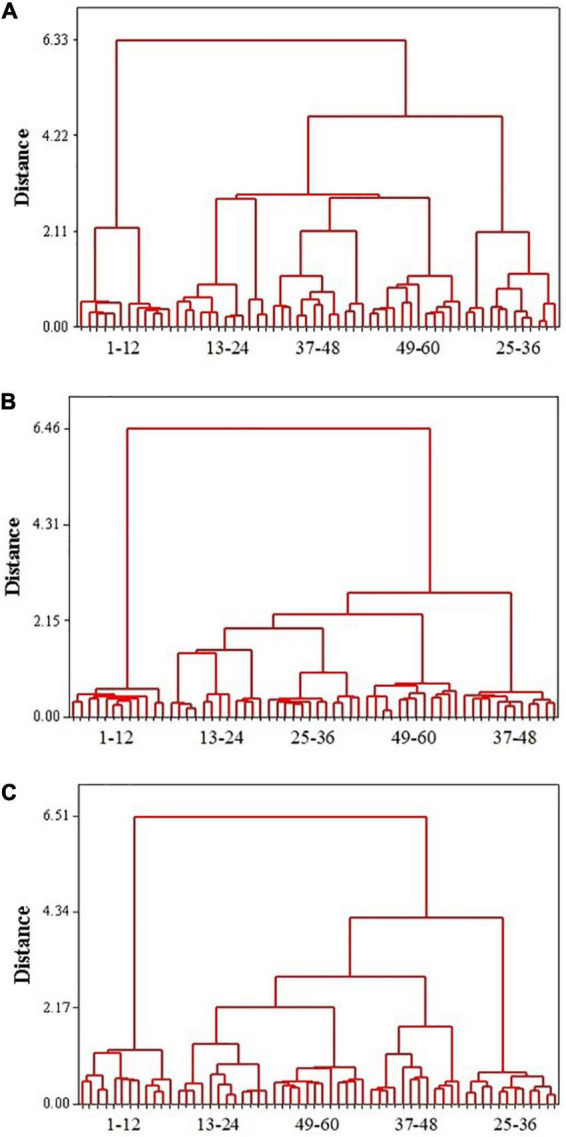
Cluster analysis of the combined e-nose and e-tongue data of seasoning powder **(A)**, seasoning mixture sauce **(B)**, and the mixture of powder and sauce **(C)**. 1–12, C brand; 13–24, D brand; 25–36, G brand; 37–48, L brand; 49–60, Z brand.

Combination of e-nose and e-tongue data has been applied in various foods analysis and quality control (31). PCA and CA were applied on the combined e-nose and e-tongue dataset to discriminate between different brands of instant starch noodles seasoning (Figures 5, 6). For the seasoning powder, seasoning mixture sauce, and the mixture of powder and sauce, the first three PCs explained 86.96, 98.09, and 94.38% of the total variance, respectively. As shown in Figure 5, all instant starch noodles seasonings were grouped into five different clusters, with no overlap among groups. Combining e-nose and e-tongue data resulted in much better clustering than occurred when analyzing either e-nose or e-tongue data alone.

Relationships between samples were visualized with a dendrogram based on a nearest neighbor method that used Euclidean distances. The combined e-nose and e-tongue data were analyzed using CA, for comparison to PCA. As shown in Figure 6, all seasoning powder samples of the same brand clustered together, with five distinct groups representing the five brands of samples. The seasoning mixture sauce and the mixture of powder and sauce similarly clustered by brand. CA gave us a preliminary classification even though the group divisions were different at different distances. The results of CA were in agreement with those from PCA, providing enough information to discriminate different brands of instant starch noodles seasonings.

### 3.6. MLPN predicted modeling

Table 2 shows the identification results of MLPN models. For the seasoning powder and seasoning mixture sauce, the identification rate of the combined data was 100% in both the training and test groups. For the mixture of powder and sauce, the identification rate of combined data was 100% in the training group. MLPN modeling demonstrated that the combination of e-nose and e-tongue data enabled better discrimination than individual e-nose and e-tongue data alone. These indicated excellent performance of classification of samples into their correct treatment groups by using the MLPN. This improvement resulted from the complementarity of e-nose and e-tongue data. The e-nose and e-tongue were sensitive to aroma and taste of instant starch noodles seasonings. This complementary behavior of cross-sensitivity plays an important role in multisensor systems (22).

**TABLE 2:** Results of multilayer perceptron neural networks analysis (MLPN) models.

Samples			Accuracy (%)					Total accuracy (%)
			***C***	***D***	***G***	***L***	***Z***	
Seasoning powder	E-nose	Train group	100.0	100.0	100.0	100.0	100.0	100.0
		Test group	100.0	100.0	100.0	100.0	100.0	100.0
	E-tongue	Train group	100.0	100.0	100.0	100.0	90.9	97.6
		Test group	100.0	100.0	100.0	100.0	100.0	100.0
	Fusion data	Train group	100.0	100.0	100.0	100.0	100.0	100.0
		Test group	100.0	100.0	100.0	100.0	100.0	100.0
Seasoning mixture sauce	E-nose	Train group	100.0	100.0	100.0	100.0	100.0	100.0
		Test group	100.0	100.0	100.0	100.0	100.0	100.0
	E-tongue	Train group	100.0	100.0	100.0	33.3	100.0	95.2
		Test group	100.0	100.0	100.0	66.7	100.0	83.3
	Fusion data	Train group	100.0	100.0	100.0	100.0	100.0	100.0
		Test group	100.0	100.0	100.0	100.0	100.0	100.0
The mixture of powder and sauce	E-nose	Train group	100.0	100.0	100.0	100.0	100.0	100.0
		Test group	100.0	100.0	100.0	100.0	100.0	100.0
	E-tongue	Train group	100.0	100.0	100.0	100.0	90.9	97.6
		Test group	100.0	100.0	100.0	100.0	100.0	100.0
	Fusion data	Train group	100.0	100.0	100.0	100.0	100.0	100.0
		Test group	100.0	100.0	100.0	66.7	100.0	83.3

## 4. Conclusion

In this paper, e-nose, e-tongue, and the combination of their data were used to discriminate instant starch noodles seasonings from five different brands. PCA explained over 85.00% of the total variance in e-nose and e-tongue data, suggested that both individual e-nose and e-tongue could discriminate different brands of instant starch noodles seasonings. For the combination data of seasoning powder, seasoning mixture sauce, and mixture of powder and sauce, the first three PCs explained 86.96, 98.09, and 94.38% of the total variance and the results of CA were in agreement with those from PCA. PCA and CA results suggested that the combination of e-nose and e-tongue showed much better classification ability than either alone. MLPN modeling demonstrated that the identification rate of the combined data was basically 100%, further indicated that the combination of e-nose and e-tongue produced more optimal discrimination and analysis of samples.

In summary, e-nose and e-tongue can provide integrated information of flavors to distinguish among samples. The combination of e-nose and e-tongue an effective method for rapid, easy, and accurate food odor analysis that is objective, highly automated and lower cost. This work may be helpful in providing some valuable information on evaluating and discriminating the quality of instant starch noodles seasoning to consumers.

## Data availability statement

The raw data supporting the conclusions of this article will be made available by the authors, without undue reservation.

## Author contributions

RM: conceptualization, investigation, writing – original draft, and data curation. HS: methodology and writing – review and editing. HC: software and formal analysis. GZ: project administration and supervision. JZ: supervision and resources. All authors contributed to the article and approved the submitted version.
